# Non-obstructive idiopathic azoospermia vs azoospermia with antecedents of cryptorchidism: ways and probabilities of becoming parents

**DOI:** 10.1186/s12610-021-00149-1

**Published:** 2021-12-09

**Authors:** Jacques Singh Sangwan, Claire Petit, Romane Sainte Rose, Cynthia Frapsauce, Laura Dijols, Jean Marc Rigot, Fabrice Guérif

**Affiliations:** 1 Service de Médecine et Biologie de la Reproduction, Hôpital Bretonneau, F-37044 Tours, France; 2 Service de Gynécologie-Obstétrique, Hôpital Robert Debré, F-37400 Amboise, France; 3 Department of Andrology and CECOS, Lille University Medical Centre, F-59000 Lille, France; 4 Université François Rabelais, F-37041 Tours, France; 5 INRAE, UMR85 PRC, CNRS, IFCE, F-37380 Nouzilly, France

**Keywords:** Non-obstructive azoospermia, Cryptorchidism, TESE-ICSI, Sperm donation, Embryo donation, Azoospermie non-obstructive, Cryptorchidie, TESE-ICSI, Don de sperme, Don d’embryons

## Abstract

**Background:**

Non-obstructive azoospermia (NOA) with history of cryptorchidism and idiopathic NOA are the most common forms of NOA without genetic aetiology. Of all patients with one of these two types of NOA, only a few will have a positive TEsticular Sperm Extraction (TESE). Of those with positive extraction followed by sperm freezing, not all will have a child after TESE-ICSI. What are the ways and probabilities of taking home a baby for patients with NOA and a history of cryptorchidism compared with patients with idiopathic NOA?

**Results:**

Patients with idiopathic NOA or NOA and a history of cryptorchidism who underwent their first TESE were included. The patients were divided into two groups: Group 1 was composed of 125 patients with idiopathic NOA and Group 2 of 55 patients with NOA and a history of surgically treated cryptorchidism. Our results showed that more than half of the NOA patients succeeded in becoming parents. The main way to fulfil their plans for parenthood is to use sperm or embryo donation (72%) for men with idiopathic NOA, whereas the majority of men with NOA and a history of cryptorchidism had a child after TESE-ICSI (58.8%).

**Conclusions:**

In our centre, before considering TESE for a patient with NOA, we explain systematically TESE-ICSI alternatives (sperm donation, embryo donation or adoption). As a result, the couple can consider each solution to become parents.

## Resume

**Contexte:**

L’azoospermie non obstructive (ANO) avec un antécédent de cryptorchidie et l’ANO idiopathique sont les causes les plus fréquentes d’ANO sans étiologie génétique. Parmi les patients présentant un de ces 2 types d’ANO, seuls quelques-uns auront une extraction positive de spermatozoïdes testiculaires (TESE). Parmi les patients ayant une extraction positive suivie d’une congélation de spermatozoïdes, tous n’obtiendront pas de naissance après TESE-ICSI. Quels sont les moyens et les probabilités de « ramener un enfant à la maison » pour les patients avec une ANO associée à un antécédent de cryptorchidie en comparaison à ceux présentant une ANO idiopathique ?

**Résultats:**

De tels patients ont été inclus dans notre étude et divisés en deux groupes : Groupe 1 composé de 125 patients avec une ANO idiopathique et Groupe 2 de 55 patients avec une ANO associée à un antécédent de cryptorchidie traitée chirurgicalement. Nos résultats ont montré que plus de la moitié des patients atteints d’ANO ont réussi à devenir parents. Le principal moyen pour réaliser leur projet parental était le recours au don de sperme ou l’accueil d’embryons (72%) pour les hommes avec ANO idiopathique, alors que la majorité des hommes avec ANO et antécédent de cryptorchidie (58.8%) achevaient leur projet parental par TESE-ICSI.

**Conclusions:**

Dans notre centre, avant d’envisager une biopsie testiculaire chez un patient présentant une ANO, les alternatives (don de sperme, accueil d’embryon ou adoption) à la TESE-ICSI sont explicitées systématiquement. En conséquence, le couple peut envisager chaque solution pour devenir parent.

## Introduction

The absence of spermatozoa in the ejaculate is identified in about 15% of infertile men. It can be classified as obstructive azoospermia and non-obstructive azoospermia (NOA). Thorough history-taking and physical examination are crucial in the classification of azoospermia etiology and may be accompanied by laboratory and genetic testing.

NOA, which comprises 60% of azoospermic men, includes several etiologies: abnormal testicular development (genetic causes, cryptorchidism, hypogonadism) and toxic exposures (radiotherapy, chemotherapy) [1, 2]. When no cause is found, it is termed idiopathic NOA. NOA with a history of cryptorchidism and idiopathic NOA are the most common forms of NOA [2]. Since the establishment of testicular sperm extraction (TESE) and the use of intracytoplasmic sperm injection (ICSI), patients with NOA have had an opportunity to conceive with their own spermatozoa [3]. According to previously published studies, the number of spermatozoa detected during a biopsy varies from 20% to 60% [4–6]. Of all patients with a positive TESE, only some will have a child after ICSI [4–6].

In the event of a negative TESE or after TESE-ICSI failure, many patients won’t be able to conceive a biological child. It is therefore crucial to inform couples of other procedures including sperm or embryo donation as well as adoption. Sperm donation is a technique used for couples suffering from severe male infertility, after repeated failures of ICSI fertilization or with a serious, transmissible genetic disorder. Embryo donation consists of embryos initially derived from intra-conjugal in vitro fertilization (IVF), whose owners no longer have parental plans and have decided to give them to other infertile couples. Embryo donation is preferentially offered to couples suffering from severe female and male infertility. The procedure of adoption in France is becoming increasingly difficult. The wait time is very long, and few couples will be able to adopt a child.

The aim of this study was to evaluate the chances of fathering a child (biological or not) in two populations of men with NOA (idiopathic or with a history of cryptorchidism).

## Materials and methods

### Patient population

This retrospective study took place in the Department of Reproductive Medicine and Biology of Tours, University Hospital, between November 2013 and December 2017 including 180 men with NOA and undergoing a conventional TESE. Azoospermia was diagnosed in all patients, due to the total absence of spermatozoa in the ejaculate of at least two sperm samples, determined by high-speed centrifugation [7]. Patients with a history of TESE were excluded.

Before surgical sperm extraction, each patient underwent a complete andrologic evaluation to determine the etiology of azoospermia. All patients underwent a thorough history-taking and physical examination including testicular volume assessment, measured manually using a Prader orchidometer. Moreover an endocrine profile (follicle stimulating hormone (FSH), inhibin B and serum testosterone levels) [8] and genetic analyses (karyotype and Y-chromosome microdeletion testing) [7] have been realized. All patients underwent an ultrasound examination of the urogenital tract and testes. The ultrasound total testicular volume (TTV) was defined as the sum of the two testis volumes [9]. NOA patients were defined as follows: bilateral hypotrophia of the testes (ultrasound testicular volume < 12 mL per testis) and elevated FSH (>10 IU/L). Idiopathic NOA was defined as NOA without previous history of cryptorchidism, without genetic aetiology (normal karyotype and absence of Yq deletions) and without etiologies that could explain NOA (radiotherapy, chemotherapy and hypogonadotropic hypogonadism).

Group 1 consisted of 125 patients with idiopathic NOA, whereas Group 2 was composed of 55 NOA patients with a history of surgically treated cryptorchidism without genetic aetiology or other antecedents that could explain the NOA.

### Ethical approval

All participating couples gave their written informed consent for the reporting and publishing of the results of the study. The protocol for this retrospective study was approved by the Ethics Committee for research involving human subjects in our hospital (Research Project No. 2016 066).

### Hormone assays

The serum FSH level was measured by chemiluminescent microparticle (CMIA) immunoassay with a normal concentration ranging from 0.9 to 10 IU/L. Serum testosterone levels were determined using CMIA with normal concentrations between 7.2 and 24.2 nmol/L. Serum inhibin B levels were measured using the enzyme immunoassay (EIA) with a detection limit of 15 pg/mL.

### Testicular sperm recovery

All the procedures of open excisional testicular bilateral biopsy (conventional TESE) were performed by the same operator and under general anaesthesia or spinal anaesthesia. The TESE procedure was performed before any ovum pick-up was carried out on the female partner. One sample was taken from each testicle; the volume of the sample was determined as a function of testicular volume [10]. A very small biopsy (approximately 1/20) was randomly taken from each specimen and examined histologically for histological diagnosis of NOA. The remaining sample was sent to the IVF laboratory for further examination, followed by spermatozoa freezing. In our centre, positive spermatozoa extraction after examination was defined by the presence of at least one spermatozoon in the testicular cell suspension. Positive extraction was followed by freezing for later use when at least one motile spermatozoon was observed in 10 fields at 400x magnification.

### IVF procedure

In cases of positive extraction followed by freezing, an ICSI was performed in the following months. The ovarian stimulation protocol used in our medical centre has already been described elsewhere [11]. After removal of cumulus–corona cells, metaphase II oocytes were injected with motile spermatozoa. Briefly, embryo culture with sequential media and assessment were carried out as follows: fertilisation (day 0) was performed in G-IVF medium™ (Vitrolife, Göteborg, Sweden). The following morning (day 1), the oocytes were individually placed in microdrops (25 μl) in G-1 PLUS medium™ (Vitrolife, Göteborg, Sweden) under Ovoil™ (Vitrolife, Göteborg, Sweden). From day 3 to day 5/6, embryo culture was performed in microdrops in G-2 PLUS medium™ (Vitrolife, Göteborg, Sweden) under Ovoil™ (Vitrolife, Göteborg, Sweden). All cultures took place in incubators at 37°C with 6% CO_2_, 5% O_2_ and 89% N_2_.

All the subsequent optical assessments were performed using an inverted microscope with Hoffman modulation contrast (x200 and x400 magnification) [12]. All observations were performed by two experienced embryologists. Embryos were evaluated 44-46 hours post-insemination/ICSI (day 2) on the basis of the number of blastomeres, shape (regularity) of cells, fragmentation rate and the presence of multinucleated blastomeres. Embryos with one or more multinucleated blastomeres were excluded from transfer and further extended embryo culture. The outcome of extended embryo culture was recorded for each individually cultured embryo. The morphological assessment was based on the expansion of the blastocoele cavity (B1 to B6) and the number and cohesiveness of the inner cell mass (ICM) and trophectodermal cells. One or two embryos with the best morphology were transferred on day 2 or day 5/6. Supernumerary blastocysts at the B2-B6 stages on day 5 or the B3-B6 stages on day 6 with an A/B inner cell mass and A/B trophectoderm were frozen for later use.

### Pregnancy follow-up

Clinical pregnancy was defined as the presence of a gestational sac with foetal heart activity on ultrasound 5 weeks after oocyte retrieval. A birth was defined as the delivery of a living infant after 20 weeks or more of gestation.

### Donation procedure

When sperm freezing was not possible or after several ICSI-TESE failures, couples were routinely offered the use of sperm or embryo donation in our centre. In order to be informed about the process of sperm and/or embryo donation, the couple meets first the surgeon, the IVF clinician and the psychologist from our IVF center. If necessary, the couple meets the biologist to discuss about the principles of donation in France, the rules of allocation and the waiting period. When sperm freezing was not possible or after several ICSI-TESE failures, 68 couples accepted the process of donation, while 37 couples refused in Group 1. In Group 2, 17 couples accepted the process of donation, while 17 couples refused.

### Outcome measure

The study ended at least 2 years after the testicular biopsy, allowing couples to carry out their parental project through other procedures (donation or adoption). The outcome measure was taking home a baby (biological or not). Thus, at the end of the study, couples completed either a biological parental project (TESE-ICSI), either a non-biological parental project (ART with a donation or adoption) or failed.

### Statistical analysis

Statistical analysis was performed using the Statview 4.1 software (Abacus Concepts, Berkeley, CA, USA). Quantitative variables were compared by analysis of variance (ANOVA) using a Student's *t*-test. The data were expressed as mean ± standard deviation (SD). Qualitative data were compared using the χ^2^ test. Fisher’s exact test was used to compare small samples. Differences were considered significant when *P* < 0.05.

## Results

### Patients

The epidemiological characteristics of patients with idiopathic NOA (Group 1) or a history of cryptorchidism (Group 2) are shown in Table 1. The values of total testicular volume (TTV), FSH, inhibin B and testosterone were similar between the two groups.

**Table 1:** Epidemiological, biological and histopathological data in Group 1 (idiopathic NOA) and Group 2 (NOA with a history of cryptorchidism) patients

All Patients (*n* = 180)	Group 1 Idiopathic NOA *n* = 125	Group 2 NOA with History of Cryptorchidism *n* = 55	***P***
Male age (years)	33.3 ± 5.9	33.5 ± 5.3	NS
Female age (years)	29.7 ± 4.1	30.3 ± 3.9	NS
Length of infertility (years)	2.9 ± 1.9	3.4 ± 2.8	NS
TTV (mL)	19.0 ± 7.0	17.8 ± 7.1	NS
FSH (IU/L)	17.0 ± 9.3	17.2 ± 10.0	NS
Inhibin B (pg/mL)	53.3 ± 74.1	57.8 ± 67.6	NS
Testosterone (nmol/L)	13.6 ± 6.2	14.0 ± 5.7	NS
Fresh examination by IVF laboratory			
Positive extraction	34 (27%)	29 (53%)	0.01
Positive extraction with freezing	32 (26%)	28 (51%)	0.01
Histological examination			
Hypospermatogenesis	49 (39%)	32 (58%)	0.028
Spermatogenesis with maturation arrest	22 (18%)	1 (2%)	0.007
Sertoli cell only	54 (43%)	22 (40%)	NS

Data are presented as mean ± SD or as percentages

*NOA* non-obstructive azoospermia, *FSH* follicle stimulating hormone, *IVF* In Vitro Fertilization, *NS* not-significant, *TTV* total testicular volume, *SD* standard deviation

### Sperm extraction

Of the 180 patients, 63(35%) had positive sperm extraction. Positive sperm extraction was significantly more frequent in Group 2 (*n*=29, 53%) than in Group 1 (*n*=34, 27%), *p* = 0.01. This result was consistent with an increased frequency of hypospermatogenesis in Group 2 compared with Group 1 (58% vs. 39%, respectively, p<0.028).

Moreover, patients with NOA and a history of cryptorchidism had almost double the probability of having a positive extraction with freezing, in comparison with idiopathic NOA patients (51% vs. 26%, respectively, *p* = 0.01).

As outlined in Table 2, on the basis of TTV, serum levels of FSH and inhibin B, we compared patients with sperm freezing and patients without sperm freezing or negative extraction. In each group, there was no significant difference when TTV, FSH and inhibin B concentrations were considered separately. Moreover, there was no significant difference for TTV, FSH or inhibin B when we combined the two groups (data not shown).

**Table 2:** Prediction of sperm extraction with freezing in patients with idiopathic NOA (Group 1) or NOA with a history of cryptorchidism (Group 2)

All Patients (*n* = 180)	Group 1 Idiopathic NOA *n* = 125			Group 2 NOA with History of Cryptorchidism *n* = 55		
	Sperm freezing	No sperm freezing^a^	*p*	Sperm freezing	No sperm freezing^a^	*p*
n	34	91		29	26	
TTV (mL)	19.6 ± 6.8	18.7 ± 7.1	NS	19.7 ± 7.0	15.8 ± 6.7	NS
FSH (IU/L)	14.9 ± 8.9	17.8 ± 9.4	NS	14.6 ± 8.9	20.2 ± 10.5	NS
Inhibin B (pg/mL)	64.7 ± 77.5	48.8 ± 72.7	NS	70.0 ± 71.9	44.0 ± 60.9	NS

^a^Negative extraction and non-freezable sperm extraction

Data are presented as mean ± SD and numbers

*FSH* follicle stimulating hormone, *NOA* non-obstructive azoospermia, *NS* not significant, *TTV* total testicular volume, *SD* standard deviation

### Different results after TESE

**Fig. 1:**
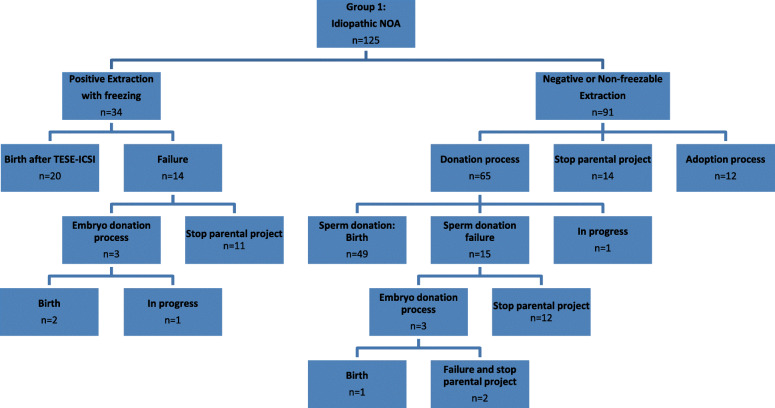
Schematic overview of patients with idiopathic non-obstructive azoospermia followed by testicular sperm extraction (TESE), until the end of the parental project

**Fig. 2:**
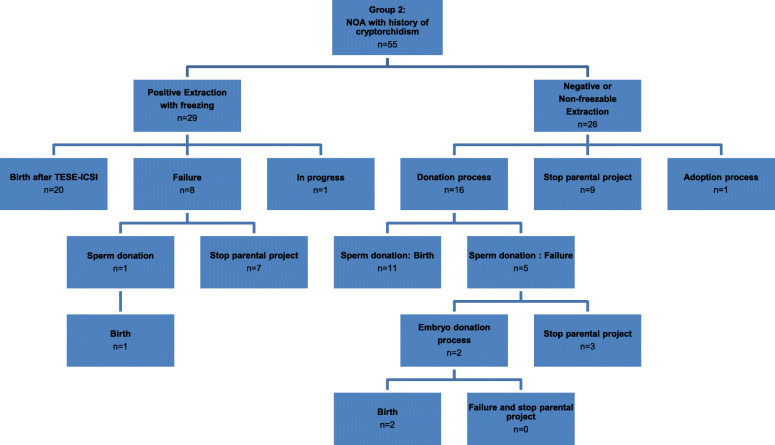
Schematic overview of patients with non-obstructive azoospermia after a history of cryptorchidism, followed by testicular sperm extraction (TESE), until the end of the parental project

In Group 1 (idiopathic NOA), all couples with positive extraction followed by sperm freezing (*n* = 34) performed ICSI cycles (Figure 1). Of the remaining 91 couples, including those with negative extraction or non-freezable sperm extraction, 26 couples (28.6%) declined the sperm or embryo donation procedure, while 65 couples (71.4%) of used a donation. In group 2 (NOA with antecedents of cryptorchidism), all couples with positive extraction followed by sperm freezing (*n*=29) underwent ICSI cycles (Figure 2). Of the remaining 26 couples with negative extraction, 10 couples (38.5%) of the declined the sperm or embryo donation procedure, while 16 couples (61.5%) accepted a donation.

### Different ways to become a father after TESE

In Group 1 (idiopathic NOA), among 99 couples undergoing an assisted reproduction procedure (ICSI with TESE, sperm or embryo donation), 72 couples (72.7%) completed a parental project (Figure 1). Successful couples became parents, mainly non-biologically, after sperm donation [49/72 (68.1%)] or embryo [3/72 (4.2%)] donation, while 27.7% of couples (20/72) became parents using the TESE procedure.

In Group 2 (NOA with antecedents of cryptorchidism), among 45 couples undergoing an assisted reproduction procedure (ICSI with TESE, sperm or embryo donation), 34 couples (75.6%) carried out their parental plans (Figure 2). In contrast to Group 1, most successful couples became parents using the TESE procedure [20/34 (58.8%)], while 41.2% (14/34) of them became non-biological parents, only after sperm [12/34 (35.3%)] or embryo [2/34 (5.9%)] donation.

Altogether, in couples undergoing an assisted reproduction procedure, the rate to take home a baby was similar in Groups 1 and 2 (72/99 (72.7%) versus 34/45 (75.6%) couples, respectively, *p*=0.37). However, in successful couples, men in Group 2 had twice the chance of becoming fathers with their own spermatozoa, in comparison with men from Group 1 [58.8% (20/34)] vs. [27.7% (20/72)], respectively, *p* < 0.01).

## DISCUSSION

NOA with a history of cryptorchidism and idiopathic NOA are the most common forms of NOA without a genetic aetiology [2]. Among such patients, not all of those who have their spermatozoa frozen after a positive TESE will have a child after TESE-ICSI [4–6]. However, these studies did not consider the possibility of carrying out a parental plan with alternative ART procedures (sperm and embryo donation) and adoption. To our knowledge, this is the first study evaluating the cumulative probability of taking home a baby, by combining different strategies after TESE.

The most common parameters considered to predict the probability of positive extraction in patients with NOA are total testicular volume, serum FSH and inhibin B levels. In patients with NOA, only a few studies have confirmed that total testicular volume could predict TESE results [13]. By contrast, several other studies have failed to confirm the predictive relationship with sufficient precision [14, 15]. Similarly, some studies have shown that serum FSH levels could be a valuable parameter [16, 17], while other studies did not support it [15]. Some studies have suggested that serum inhibin B could be a useful parameter alone [16, 18, 19] or in combination with serum FSH [20]. However, the serum level of inhibin B was not found to be sufficiently discriminating by other studies [15]. In our study, we found no significant difference between patients with a positive TESE followed by sperm freezing and those with a negative extraction or non-freezable sperm extraction. Previous studies analyzing biological or hormonal markers to predict sperm extraction have often larger populations allowing to reach statistical differences. In our study, as our groups are strictly defined, the size of the population is small. It could be the major explanation leading to the lack of difference about such biological parameters.

It seems that there was no consensus defining a limit below which it would be superfluous to freeze sperm from TESE, because the chances of pregnancy would be very low. In a previous study, a testicular cell suspension obtained after a wet laboratory preparation was frozen for later use when at least one sperm was observed [21]. Of their patients with NOA, 41% had a positive TESE with a cumulative crude delivery rate of 37% after 6 ICSI. In our IVF centre, we applied stricter criteria for freezing after TESE. According to this threshold, testicular spermatozoa were detected in 35% (63/180) of patients with NOA. Among our patients with frozen sperm after TESE, the cumulative delivery rate after 4 potential ICSI cycles was 66.7% (40/60). The difference in the cumulative delivery rate between our study and that of Vloeberghs *et al.* could be explained by our stricter criteria for freezing testicular sperm. In our centre, back-up TESE (i.e. rescue TESE performed on the day of oocyte retrieval if frozen–thawed suspensions could not be used) was not included in the sperm exploration strategy. However, our stricter freezing criteria reduced the risk of unnecessary oocyte retrieval due to the lack of sperm available for ICSI without decreasing the chances of achieving a birth after TESE.

Several studies have shown that NOA patients with a history of cryptorchidism are more likely to have positive sperm retrieval during TESE than men with idiopathic NOA [13, 22, 23]. Our results are consistent with such observations. In fact, in our study, NOA patients with a history of cryptorchidism had almost double the chances of having a positive extraction followed by freezing, in comparison with idiopathic NOA patients (51% vs. 26%, respectively).

When sperm extraction was positive after TESE, 63.5% of couples (40/63) managed to have a baby using TESE-ICSI and only 6.3% through a donation process after failure of TESE-ICSI (4/63). When sperm extraction was negative after TESE, 53.8% of couples (63/117) managed to have a baby only through the donation of sperm or embryos. The originality of our study was that it highlighted all the procedures for becoming a father (including ART and non-ART procedures) available to NOA patients, regardless of the results of TESE explorations. Indeed, biological paternity is not the only way to conceive a child. Of all NOA couples included in our study, 58.9% (106/180) took home a baby, mainly after sperm or embryo donation (36.7% = 66/180) and less frequently using intra-conjugal spermatozoa (22.2% = 40/180). Around seven percent of couples (13/180) initiated an adoption process. When patients with idiopathic NOA and those with a history of cryptorchidism were analysed separately, we observed that patients with antecedents of cryptorchidism had approximately double the frequency of positive extraction followed by freezing. Altogether, such results allowed them to become fathers with their own spermatozoa more frequently in comparison with idiopathic NOA patients (36.4% vs. 16.0%, respectively), and there was no difference between the two groups in the overall rate of taking home a baby 61.8% vs. 57.6%, respectively), when alternative procedures were also considered.

Failure of sperm extraction after testicular biopsy or failures of pregnancy after TESE-ICSI are dramatic events for couples. All couples followed in our center for infertility with azoospermia have different consultations with our clinician andrologist, our psychologist and an IVF physician in order to prepare the course of IVF and possibly the use of sperm donor. Indeed, even after these times of explanations and exchanges, sperm donation procedure for parenthood is experienced very differently by couples treated for infertility with azoospermia. Reasons are mainly psychological. Some explanations put forward by couples (both men and women) are: the loss of "transmission of the genetic heritage", fear of the family gaze, feeling of adultery through the technique of sperm donation. Religious reasons are also sometimes put forward. However, often the couple concludes not to use donation without indicating clearly what the reasons are for this. We did not note any differences in the reasons given by the couple refusing recourse to donation after TESE failure or ICSI-TESE failure.

In embryo donation although the intented mother carries the baby until the birth, neither the woman or the man from the recipient couple will be genetically related to the resulting child. Thus, from a genetic point of view, families issued from embryo donation do resemble to families issued from adoption. However, legally, practically and psychosocially, there are differences between embryo donation and adoption. A key question for such couples is how to tell to their child the mode of conception which is fundamentally different between both procedures. For some parents, before planning to disclose in the future, their child should have first some knowledges about physiological reproduction. Professionals from Assisted reproduction technologies as well as form adoption procedure, initially advocated secrecy. However, a spirit of openness has emerged among professionals of assisted reproduction.

Some couples refuse the use of sperm or embryo donation to fulfill their parental project, while other split up after the announcement of such failures. In such situation, the medical staff has to inform the couple that adoption, which is a non-medical way to become parents, could be considered. However, the final decision is in the hands of the affected couple. Adoption has always been a long process. Over the past years, it tended to become more and more complex. Thus, in France, the number of adoptable children decreases years after years. In addition, international adoption had also become more difficult. Many countries have stopped the possibility of adoption of children by foreigners. Altogether, fewer and fewer couples manage to become parents by this procedure or only after increasingly long delays. In the field of the medical reproduction, the scientific literature on adoption is poor. For all this reasons (refusal of donation, split up, non-medical project of parenthood) couple are less inclined to answer to medical solicitation. In such conditions, the follow up of unsuccessful couples after initiation of an ART procedure remains quite difficult.

## Conclusions

In our study, more than half of patients with NOA became parents. The majority of these patients were successful with the help of a donation and not by TESE-ICSI. Men with a history of cryptorchidism were twice more likely to become fathers with their own spermatozoa than men with idiopathic NOA. In our IVF centre, before considering TESE for a patient with NOA, we systematically explain the alternatives of TESE-ICSI (sperm donation, embryo donation or adoption) and the expected results from these processes. As a result, these couples can choose how they want to try to become parents.

## Data Availability

The datasets used and analysed during the current study are available from the corresponding author on reasonable request.

## Abbreviations

ART: Assisted reproductive technology
CMIA: Chemiluminescent microparticle
EIA: Enzyme immunoassay
FSH: Follicle-stimulating hormone
ICSI: Intra-cytoplasmic sperm injection
IVF: In vitro fertilization
NOA: Non-obstructive azoospermia
TESE: Testicular sperm extraction
TTV: Total testicular volume
